# Shift work characteristics and burnout among nurses: cross-sectional survey

**DOI:** 10.1093/occmed/kqad046

**Published:** 2023-05-02

**Authors:** C Dall’Ora, O-Z Ejebu, J Ball, P Griffiths

**Affiliations:** School of Health Sciences, University of Southampton, Southampton, UK; National Institute for Health Research Applied Research Collaboration, Wessex SO16 7NP, UK; School of Health Sciences, University of Southampton, Southampton, UK; National Institute for Health Research Applied Research Collaboration, Wessex SO16 7NP, UK; School of Health Sciences, University of Southampton, Southampton, UK; School of Health Sciences, University of Southampton, Southampton, UK; National Institute for Health Research Applied Research Collaboration, Wessex SO16 7NP, UK

## Abstract

**Background:**

Nurses working long shifts (≥12 h) experience higher levels of burnout. Yet other shift characteristics, including fixed versus rotating night work, weekly hours and breaks have not been considered. Choice over shift length may moderate the relationship; however, this has not been tested.

**Aims:**

To examine the association between shift work characteristics and burnout and exhaustion, and whether choice over shift length influences burnout and exhaustion.

**Methods:**

Cross-sectional online survey of nursing staff working in the UK and Ireland. We recruited two large National Health Service Trusts, through trade union membership, online/print nursing magazines and social media. We assessed associations using both univariable and multivariable generalized linear models.

**Results:**

We had 873 valid responses. Reports of inadequate staffing levels (odds ratio [OR] = 2.84; 95% confidence interval [CI] 2.08–3.90) and less choice over shift length (OR = 0.20; 95% CI 0.06–0.54) were associated with higher burnout in multivariable models. Similar associations were found for exhaustion, where rarely or never taking breaks was also a predictor (OR = 1.61; 95% CI 1.05–2.52). Nurses who worked long shifts had less choice than those working shifts of 8 h or less (66% of 12-h shift nurses versus 44% 8-h shift nurses reporting having no choice), but choice did not moderate the relationship between shift length and burnout and exhaustion.

**Conclusions:**

The relationship between long shifts and increased burnout reported previously might have arisen from a lack of choice for those staff working long shifts. Whether limited choice for staff is intrinsically linked to long shifts is unclear.

## Teaser

Burnout is an occupational phenomenon affecting many healthcare workers. Long shifts are associated with burnout, but it is unclear if this adverse effect is apparent in those that actively choose to work this shift pattern. Our survey of 873 nurses found that having choice decreases the likelihood of experiencing burnout. Nurses working long shifts were less likely to have choice and more likely to report adverse working environments.

**Key learning points**

**What is already known about this subject:**

- The relationship between long shifts and burnout and exhaustion in nurses has been confirmed by several studies, but previous studies did not consider other shift work characteristics and the degree of choice around shift length.

**What this study adds:**

- Adjusting for other shift work characteristics and degree of choice, the association between shift length and burnout and exhaustion was not statistically significant.
- Lower degree of choice and inadequate staffing levels were associated with a higher risk of burnout and exhaustion.
- Nonetheless, nurses working long shifts reported the lowest degree of choice and worst staffing levels.

**What impact this may have on practice or policy:**

- More choice and flexibility around work hours may reduce burnout and exhaustion, but without tackling negative work environments, choice is unlikely to be an effective solution.

## Introduction

Nursing as an occupational sector often involves shift work and long working hours. This means nurses are at a high risk of being affected by occupational stress and burnout [1, 2]. Burnout is an occupational phenomenon, caused by prolonged exposure to physical and psychological occupational hazards, specifically poor work environments [3]. It is a state characterized by severe exhaustion, cynicism, and impaired cognitive and emotional functioning [4]. The prevalence of burnout among nurses working in Europe and the USA has been reported to range between 25% and 37% [5, 6]. The consequences of burnout and exhaustion are serious for both staff and their employers. When burnt out, workers are more likely to experience sickness absence and intention to leave [7]. Nurse burnout also impacts patient safety as it is associated with increased risk of medication errors, and patients are more likely to experience infections, falls and adverse events [1].

The association between aspects of shift work for nurses, in particular, long shifts of 12 h or more, and burnout has been highlighted by several studies. Early reports of nurses working 12-h shifts being more likely to experience burnout compared to their counterparts working 8-h shifts from early studies with small samples [8] have been supported by more recent and robust studies. Large cross-sectional surveys of thousands of hospital-registered nurses found an association between 12-h shifts and the emotional exhaustion dimension of burnout [6, 9]. Not all studies support this association. A survey of 805 nurses found that working in units deploying 12-h shifts was associated with lower emotional exhaustion [10]. In this study, nurses who worked 12-h shifts had chosen to do so for the duration of the study. Theories of burnout indicate that such choice has the potential to modify the relationship between shift characteristics and burnout, including the Job Demands-Resources model [11]. According to this model, long shifts are job demands, leading to burnout, whereas choice over shift length indicates autonomy, characterized as a job resource, potentially moderating the adverse effect of demands. Choice around shift patterns has been associated with improved health and sleep, and reduced fatigue for employees [12], but this has not been explored in nursing.

Studies with small samples have shown an association between night working and burnout [13], although this relationship has the potential to be shaped by how night shifts are worked, whereby permanent and rotating shifts might have different effects on nurses’ health and well-being [14]. Nonetheless, schedule rotation has not been considered in relation to burnout in nursing. Working overtime and long weekly hours have also been associated with burnout, although the evidence is scant [15]. The evidence gap around the effect of shift work characteristics other than shift length on burnout in nursing is apparent. In addition, previous analyses failed to control for other important job demands, including workload and ability to rest during the shift. Therefore, the aim of this study is to examine the association between shift work characteristics and burnout, and whether choice over shift length influences burnout.

## Methods

This was a cross-sectional survey of nursing staff providing care (registered nurses, nursing associates and healthcare support workers).

We launched the survey online from June to October 2021 via two routes: (i) To a targeted population of nursing staff at two large Mental Health & Community National Health Service (NHS) Trusts in the Wessex region (England). Both Trusts are geographically dispersed and comprise several hospital sites. Local principal investigators disseminated a link to the survey and a QR code to offer flexibility around the completion of the survey. (ii) Through an open invitation via social media, authors’ and their institutions’ Twitter accounts; nursing magazines (Nursing Times and Nursing Standard); membership lists of Unison (the UK’s largest trade union) and the Royal College of Nursing Library page.

Any nurse providing care in the UK or Ireland was eligible to participate. Student nurses and nurses working in administrative, research, academic or managerial positions not involving clinical care provision were excluded. Due to multiple recruitment channels with uncertain audience numbers, it was not possible to estimate an exact sample size, and we did not undertake any power calculations. We aimed to recruit 800 or more participants, more than sufficient to give 10–20 observations per variable in multiple regression models, a widely suggested minimum sample size [16].

The questionnaire was developed with advice and feedback from nursing unions’ leads, two registered nurses and a patient. It was pilot tested and adjusted with two registered nurses, one healthcare assistant and one nurse manager.

Burnout was the primary outcome. Although there is considerable disagreement about a precise definition [4], burnout was defined in International Classification of Diseases 11th (ICD11) as a syndrome arising from chronic workplace stress characterized by feelings of energy depletion or exhaustion; increased mental distance or feelings of negativism or cynicism related to one’s job; and reduced professional efficacy [17]. We measured burnout with the Burnout Assessment Tool (BAT)—short version which comprises 12 items and 4 dimensions: exhaustion, mental distance, cognitive impairment and emotional impairment [18]. In contrast to the Maslach Burnout Inventory [19], the BAT can provide a single burnout score. Because some definitions of burnout focus only on exhaustion [3] and much nursing literature reports only emotional exhaustion [9,20], we used the BAT exhaustion dimension as a secondary outcome. According to the BAT manual, we classified participants as experiencing burnout if they scored 2.54 or more on the BAT total score; exhaustion was determined by a score of 3.17 or more on the BAT Exhaustion dimension [18].

Shift work variables were those identified by Dall’Ora et al. in their literature review [21]: shift length, timing of work, rest opportunities, weekly working hours and overtime. We asked respondents about their shift characteristics considering their typical working week. We categorized shift length into three categories: ≤8; >8–<12; ≥12 h. Participants were categorized into the following types of shift pattern: no shift work (i.e. standard office hours), day only (including evening), rotating (including night) and permanent night. We calculated the total weekly hours from nurse-reported contracted hours and mean weekly paid and unpaid extra work hours. We categorized the total weekly hours into <37.5 as part time, between 37.5 and 48 as full time, and more than 48 as excessive weekly hours (in UK, 48 h is the legal limit but given staff shortages, it is likely to occur).

To capture workload and time pressure as per the Job Demands-Resources model [11], we collected data around staffing adequacy and ability to take breaks during shifts. To capture the degree of choice around shift length, we asked: ‘To what extent are you able to choose your shift length?’ with responses on a five-item Likert scale (not at all; a little; to some extent; a large extent and completely). We also collected demographic data including age and gender identity.

We used generalized linear models to examine the association between shift characteristics, including shift length category, type of shift pattern (i.e. no shift work; day only; rotating with night and permanent night), total weekly hours of work, controlling for choice over shift length, frequency of break taking during the shift and staffing adequacy. To assess whether the degree of choice over shift length acted as a moderator in the relationship between shift length and burnout, we added an interaction term between shift length and choice to our model. We replicated this in the model with exhaustion as outcome. We selected our final models based on the inclusion of all variables identified as relevant to burnout in the Job Demands-Resources model, with other demographic and workplace variables included only if they improved model fit according to the Akaike information criterion and Bayesian information criterion.

Ethical approval was obtained from the University of Southampton, Faculty of Environmental and Life Sciences Ethics Committee (Approval ID: 65122.A2 and 57489.A2).

## Results

We received 976 responses to our survey, 873 via social media and unions and nursing magazines’ membership lists and 83 via the two Trusts. After removing invalid responses (i.e. not nursing staff, not working in the UK or Ireland), the final sample comprised 873 nursing staff. Of these, 658 (75%) were registered nurses, 188 (22%) were healthcare assistants and 27(3%) were nursing associates. Mean age was 42, with a standard deviation of 12 and 752 (86%) identified as female. Frequencies of specific gender identities (i.e. male, gender neutral, gender fluid, non-binary and trans) are not reported due to small numbers in each cluster and residual risk of identification despite anonymity. Table 1 summarizes the sectors, settings and areas of practice of respondents, the majority working for the NHS in hospital inpatient wards.

**Table 1.** Sector, setting and area of practice of surveyed nurses

Sector	*N* (%)
NHS	805 (93)
Private healthcare	43 (5)
Social care	19 (2)
Setting	
Hospital inpatient ward	584 (66)
Other hospital unit/role - e.g. day surgery, outpatients, outreach	129 (16)
Community	87 (9)
Primary care	36 (5)
Care home/ nursing home	31 (4)
Area of practice	
Acute adult care	334 (40)
Critical care	89 (10)
District nursing	28 (3)
End of life care	20 (2)
Mental Health	144 (17)
Occupational Health	19 (2)
Older people care	62 (7)
Paediatrics	49 (6)
Rehabilitation	53 (6)
Maternity and women health	16 (2)
Other^a^	34 (4)

^a^Other includes Blood donation = 6; Health visiting = 5; Practice Nursing = 8; School nurse = 2; Learning Disabilities = 13.

The majority reported working shifts of 12 h or longer (*n* = 558, 65%), and 211 (24%) worked 8 h or shorter shifts. The remaining 96 (12%) worked shifts longer than 8 h and shorter than 12 h. The majority worked rotating shift patterns including night shifts (*n* = 443, 52%), while 53(6%) worked permanent night shifts. Many (*n* = 411, 50%) reported working between 37.5 h/week and the 48-h threshold of the European Working Time directive and the UK Working Time Regulations. These directives and regulations state that employers cannot compel employees to work more than 48 h/week [22, 23], but almost one third of participants (*n* = 229, 28%) reported working above 48 h/week.

The distribution of degree of choice and other shift and workload variables by shift length is reported in Table 2. A minority reported having a large degree or complete choice over their shift length. The majority working ≥12-h shifts reported having no choice at all, compared to nurses working ≤8-h shifts. Nurses working ≥12-h shifts were also working a high number of weekly hours, with 32% working ≥12-h shifts reporting more than 48 h/week versus 16% working ≤8-h shifts. Higher proportions of nurses working long shifts reported inadequate staffing levels than their counterparts working short shifts (63% versus 50%).

**Table 2.** Shift work and workload variables by shift length category

	Shift category			
	≤8 h, *n*(%)	>8–<12 h, *n*(%)	≥12 h, *n*(%)	All, *n*(%)
**Degree of choice around shift length**				
None	88 (17)	53 (11)	366 (72)	507 (100)
A little	43 (31)	12 (9)	85 (60)	140 (100)
To some extent	44 (32)	18 (17)	54 (51)	106 (100)
A large extent	24 (39)	7 (11)	31 (50)	62 (100)
Complete	8 (28)	4 (14)	16 (57)	28 (100)
Total^a^	207	94	552	853
**Work schedule**				
No shift work	82 (73)	29 (25)	2 (2)	113 (100)
Day only (including evening)	76 (30)	34 (13)	143 (57)	253 (100)
Rotating (including night)	49 (11)	19 (4)	375 (85)	443 (100)
Permanent night	2 (4)	14 (26)	37 (70)	53 (100)
Total^a^	209	96	557	862
Total weekly hours (including overtime)				
<37.5 (part time)	69 (37)	22 (12)	93 (51)	184 (100)
Between 37.5 and less than 48	102 (25)	38 (9)	271 (66)	411 (100)
≥48 (above EU Work Time directive)	27 (12)	30 (13)	172 (75)	229 (100)
Total^a^	198	90	536	824
Ability to take breaks during shifts				
Always or usually	96 (22)	44 (10)	304 (68)	444 (100)
Sometimes	46 (21)	23 (11)	144 (68)	213 (100)
Rarely or never	68 (33)	28 (14)	110 (53)	206 (100)
Total^a^	210	95	558	863
Staffing levels				
Adequate	105 (30)	34 (10)	208 (60)	347 (100)
Inadequate	106 (20)	60 (12)	350 (68)	516 (100)
Total^a^	211	94	558	863

^a^Total number of responses differs for some variables due to invalid or missing responses.

Over half, 497 (57%) were classified as experiencing burnout. The majority were classified as experiencing high exhaustion (*n* = 581, 67%). Table 3 displays the distribution of burnout and exhaustion by shift length category.

**Table 3:** Burnout and exhaustion by shift length category

	Shift category		
	≤8 h, *n*(%)	>8–<12 h, *n*(%)	≥12 h, *n*(%)
No burnout	109 (51)	28 (30)	234 (42)
Burnout	102 (49)	67 (70)	324 (58)
All	211 (100)	95 (100)	558 (100)
No exhaustion	91 (43)	28 (30)	166 (30)
Exhaustion	120 (57)	67 (70)	392 (70)
All	211 (100)	95 (100)	558 (100)

Univariable associations between long shifts and burnout were statistically significant in the generalized linear model (see Table 4). When controlling for other shift characteristics, staffing adequacy and degree of choice, all shifts longer than 8 h were associated with a higher likelihood of experiencing burnout, although only results for shifts between 8 and 12 h were statistically significant. Having complete degree of choice of shift length compared to having no choice at all was associated with a reduced likelihood of experiencing burnout. Inadequate staffing levels were also statistically significantly associated with burnout. Having fewer break opportunities and working full time and above, the weekly hour limit was also associated with higher odds of burnout, albeit not statistically significant.

**Table 4:** Associations between shift work, workload variables, degree of choice, and burnout and exhaustion

	Burnout				Exhaustion			
	OR	95% CI	aOR	95% CI	OR	95% CI	aOR	95% CI
Shift length category								
≤8 h (reference category)								
>8–<12 h	**2.55** ^*^	**2.53–4.33**	**2.10** ^*^	**1.18**–**3.80**	**1.81** ^*^	**1.08–3.08**	1.43	0.79–2.62
≥12 h	**1.47** ^*^	**1.07–2.03**	1.04	0.66–1.62	**1.79** ^*^	**1.28–2.48**	1.24	0.78–1.96
Work schedule								
No shift work (reference category)								
Day only (including evening)	0.93	0.59–1.45	0.93	0.54–1.60	1.12	0.70–1.76	1.11	0.63–1.95
Rotating (including night)	1.32	0.87–2.00	1.25	0.71–2.20	**1.59** ^*^	**1.03–2.44**	1.32	0.74–2.37
Permanent night	1.03	0.53–1.99	0.74	0.33–1.69	1.40	0.71–2.83	1.30	0.55–3.16
Total weekly hours								
<37.5 (part time) (reference category)								
Between 37.5 and less than 48	1.41	0.99–2.00	1.19	0.80–1.76	**1.59** ^*^	**1.11–2.28**	1.22	0.81–1.84
≥48 (above EU Work Time directive)	**1.64** ^*^	**1.11–2.43**	1.22	0.78–1.90	**1.89** ^*^	**1.26–2.86**	1.24	0.77–2.00
Ability to take breaks during shifts								
Always or usually (reference category)								
Sometimes	**1.56** ^*^	**1.12–2.18**	1.20	0.83–1.76	**1.63** ^*^	**1.15–2.32**	1.17	0.79–1.76
Rarely or never	**1.66** ^*^	**1.18–2.33**	1.15	0.78–1.71	**2.13** ^*^	**1.47–3.11**	**1.61** ^*^	**1.05–2.52**
Staffing levels adequacy								
Adequate (reference category)								
Inadequate	**3.13** ^*^	**2.37–4.17**	**2.84** ^*^	**2.08–3.90**	**3.59** ^*^	**2.68–4.84**	**2.86** ^*^	**2.06–3.99**
Degree of choice around shift length								
None (reference category)								
A little	**0.52** ^*^	**0.35–0.76**	**0.55** ^*^	**0.36–0.83**	**0.50** ^*^	**0.34–0.74**	**0.55** ^*^	**0.35–0.85**
To some extent	**0.60** ^*^	**0.40–0.90**	0.66	0.42–1.04	**0.38** ^*^	**0.25–0.58**	**0.44** ^*^	**0.28–0.70**
A large extent	**0.42** ^*^	**0.24–0.71**	0.56	0.30–1.02	**0.31** ^*^	**0.18–0.52**	**0.42** ^*^	**0.23–0.77**
Complete	**0.14** ^*^	**0.05–0.34**	**0.20** ^*^	**0.06–0.54**	**0.12** ^*^	**0.04–0.27**	**0.10** ^*^	**0.03–0.29**

Abbreviation: aOR, adjusted odds ratio. Bold results are statistically significant at *P* < 0.05.

^*^Statistically significant at *P* < 0.05.

Similar to burnout, in univariable models, shifts of 12 h or longer were associated with exhaustion. Nurses working ≥12-h shifts were more likely to experience exhaustion than those working ≤8-h shifts, but the relationship was not statistically significant. Similarly, work schedules involving shift work, working 37.5 h/week or more, reduced ability to take breaks, perceived inadequate staffing levels were all associated with a higher likelihood of experiencing burnout, with taking breaks rarely or never and inadequate staffing levels being the only statistically significant associations. The higher the degree of choice, the lower the burnout. All unadjusted and adjusted odds ratios and confidence intervals are reported in Table 4.

To determine if there was moderation, we tested models with an interaction between shift length and choice, but the interaction was not statistically significant, and it did not improve model fit.

## Discussion

We found that working shifts between 8 and 12 h, inadequate staffing levels and having no choice over shift length were associated with an increased risk of burnout. Inadequate staffing levels, no choice over shift length and rarely or never taking breaks were associated with exhaustion. Neither burnout nor exhaustion was significantly associated with long shifts, type of work schedule or weekly working hours in multivariable models.

This was the first study to examine how shift work characteristics, including choice over shift length, influence burnout in nursing staff. However, our study has some limitations. Our sample was relatively small compared to some other surveys that explored nursing shift length [24], but larger than other cross-sectional UK nursing surveys conducted simultaneously [25]. Also, online survey participants self-select, and our sharing mechanism through social media and nursing magazines’ readership lists meant that we could not control its distribution. It is possible that those experiencing burnout or dissatisfaction related to their shift pattern were more likely to participate. However, our selection mechanism cannot explain our observed associations between working conditions and burnout although observed relationships could be attenuated. The COVID-19 pandemic probably affected work patterns, workload and burnout for our respondents, so our findings are likely to reflect this. Lastly, our study relied on cross‐sectional data, so we could not assess the prolonged exposure to adverse work characteristics, although we asked staff to answer questions considering their typical working week. No causal relationship between shift characteristics, staffing adequacy, and choice and burnout can be concluded. The subjective nature of the data makes our findings susceptible to common methods variance [26].

Twelve-hour shifts were associated with increased burnout, and especially exhaustion in the multivariable models, although the association was not significant. Given the results of previous studies, it is possible that this result could be a consequence of our smaller sample size compared to previous studies [6,8,9]. Also, these studies did not account for choice over shift length. It is possible that this relationship was confounded by choice, as including choice in our models attenuated the effect of shift length. While any degree of choice reduced burnout, there was no linear association with increasing choice and those reporting complete choice showed the lowest positive association with burnout. The absence of a dose–response should reduce confidence in a causal interpretation, although absolute numbers reporting complete choice were very low. We don’t know how respondents interpreted the response options, and certainly, the effect of varying degrees of choice remains uncertain. We did not find that choice acts as a moderator in the relationship between shift length and burnout. This is in line with the increasing literature on the beneficial effects of self-rostering and having higher control over one’s working hours [27, 28]. When given choice over work hours, employees are more likely to be satisfied and stay in their jobs [29].

While this finding could be interpreted as addressing the widespread concern over the adverse effects of long shifts, nurses working long shifts reported less choice. The reason for this relationship is unclear. It is possible that implementing long shifts removes or reduces choice or that such shifts are simply not preferred by many staff, leading to more reports of lack of choice.

Shift length was also related to other variables that were statistically significant predictors of burnout in our multivariable models. Those working 12-h shifts were more likely to perceive their staffing levels to be inadequate; nurses working long shifts are exposed to worse working conditions altogether, compared to their counterparts working shorter shifts. Nurses working shifts of 12 h or longer also reported fewer break opportunities than nurses working 8-h shifts or shorter. Why shift length is associated with these factors is unclear, and it is possible that long shifts may enable these adverse working practices. For example, there is no overlap between shifts to allow staff to take breaks. Long shifts were promoted under the assumption they would maintain nurse-to-patient ratios with fewer total staff despite evidence that staff productivity may be lowered during such shifts (and so net staffing resource lowered) [30]. We found that working shifts between 8 and 12 h were associated with higher burnout in multivariable models. The reason for this is unclear and worthy, of further investigation although the number working this shift length was small *n*(10%).

Our finding that staff who had a complete choice over shift patterns were less likely to experience burnout and exhaustion should be considered carefully. Complete choice over work hours may have little impact if other factors contributing to burnout and exhaustion persist. Also, complete choice of work hours might be impractical in settings providing 24/7 care. Innovative solutions that balance nurses’ preferences and health services’ staffing needs while limiting unhealthy working hours may improve nurses’ burnout and exhaustion. Given the implications of burnout on nurse well-being, retention and patient safety, finding such solutions is imperative.

## Data Availability

Data collected through an open invitation online are available at: https://doi.org/10.5258/SOTON/D2278

## References

[1] Dall’Ora C , Ball J, Reinius M, Griffiths P. Burnout in nursing: a theoretical review. Hum Resour Health 2020;18:41. 32503559 10.1186/s12960-020-00469-9 PMC7273381

[2] Wisetborisut A , Angkurawaranon C, Jiraporncharoen W, Uaphanthasath R, Wiwatanadate P. Shift work and burnout among health care workers. Occup Med (Lond) 2014;64:279–286. 24550196 10.1093/occmed/kqu009

[3] Guseva Canu I , Marca SC, Dell’Oro F et al. Harmonized definition of occupational burnout: a systematic review, semantic analysis, and Delphi consensus in 29 countries. Scand J Work Environ Health 2021;47:95–107. 33258478 10.5271/sjweh.3935 PMC8114565

[4] Schaufeli W. The burnout enigma solved? Scand J Work Environ Health 2021;47:169–170. 33604675 10.5271/sjweh.3950 PMC8126437

[5] McHugh MD , Kutney-Lee A, Cimiotti JP, Sloane DM, Aiken LH. Nurses’ widespread job dissatisfaction, burnout, and frustration with health benefits signal problems for patient care. Health Aff (Millwood) 2011;30:202–210. 21289340 10.1377/hlthaff.2010.0100 PMC3201822

[6] Dall’Ora C , Griffiths P, Ball J, Simon M, Aiken LH. Association of 12 h shifts and nurses’ job satisfaction, burnout and intention to leave: findings from a cross-sectional study of 12 European countries. BMJ Open 2015;5:e008331. 10.1136/bmjopen-2015-008331 PMC4577950 26359284

[7] Jourdain G , Chênevert D. Job demands–resources, burnout and intention to leave the nursing profession: a questionnaire survey. Int J Nurs Stud 2010;47:709–722. 20138278 10.1016/j.ijnurstu.2009.11.007

[8] Iskera-golec I , Folkard S, Marek T, Noworol C. Health, well-being and burnout of ICU nurses on 12- and 8-h shifts. Work Stress 1996;10:251–256.

[9] Stimpfel AW , Sloane DM, Aiken LH. The longer the shifts for hospital nurses, the higher the levels of burnout and patient dissatisfaction. Health Aff (Millwood) 2012;31:2501–2509. 23129681 10.1377/hlthaff.2011.1377 PMC3608421

[10] Stone PW , Du Y, Cowell R et al. Comparison of nurse, system and quality patient care outcomes in 8-hour and 12-hour shifts. Med Care 2006;44:1099–1106. 17122714 10.1097/01.mlr.0000237180.72275.82

[11] Demerouti E , Bakker AB, Nachreiner F, Schaufeli WB. The job demands-resources model of burnout. J Appl Psychol 2001;86:499–512. 11419809

[12] Shifrin NV , Michel JS. Flexible work arrangements and employee health: a meta-analytic review. Work Stress 2022;36:60–85.

[13] Vidotti V , Ribeiro RP, Galdino MJQ, Martins JT. Burnout syndrome and shift work among the nursing staff. Rev Lat Am Enfermagem 2018;26:e3022. 30110099 10.1590/1518-8345.2550.3022 PMC6091368

[14] Chang W-P , Peng Y-X. Influence of rotating shifts and fixed night shifts on sleep quality of nurses of different ages: a systematic literature review and meta-analysis. Chronobiol Int 2021;38:1384–1396. 34056959 10.1080/07420528.2021.1931273

[15] Ilhan MN , Durukan E, Taner E, Maral I, Bumin MA. Burnout and its correlates among nursing staff: questionnaire survey. J Adv Nurs 2008;61:100–106. 18034813 10.1111/j.1365-2648.2007.04476.x

[16] Jenkins DG , Quintana-Ascencio PF. A solution to minimum sample size for regressions. PLoS One 2020;15:e0229345. 32084211 10.1371/journal.pone.0229345 PMC7034864

[17] World Health Organization. *Burn-Out: An ‘Occupational Phenomenon’: International Classification of Diseases* . 2019. https://www.who.int/news/item/28-05-2019-burn-out-an-occupational-phenomenon-international-classification-of-diseases (2 February 2023, date last accessed).

[18] Schaufeli WB , Desart S, De Witte H. Burnout Assessment Tool (BAT)—development, validity, and reliability. Int J Environ Res Public Health 2020;17:94959495. 10.3390/ijerph17249495 PMC7766078 33352940

[19] Maslach C , Jackson SE, Leiter M. Maslach Burnout Inventory Manual. 4th edn. Mind Garden, Inc.; 2016.

[20] Aiken LH , Clarke SP, Sloane DM, Sochalski J, Silber JH. Hospital nurse staffing and patient mortality, nurse burnout, and job dissatisfaction. JAMA 2002;288:1987–1993. 12387650 10.1001/jama.288.16.1987

[21] Dall’Ora C , Ball J, Recio-Saucedo A, Griffiths P. Characteristics of shift work and their impact on employee performance and wellbeing: a literature review. Int J Nurs Stud 2016;57:12–27. 27045561 10.1016/j.ijnurstu.2016.01.007

[22] Directive 2003/88/EC of the European Parliament and of the Council of 4 November 2003 Concerning Certain Aspects of the Organisation of Working Time, L 299 (2003).

[23] Working Time Regulations SI 1998/1833 (1998).

[24] Ball J , Day T, Murrells T et al. Cross-sectional examination of the association between shift length and hospital nurses job satisfaction and nurse reported quality measures. BMC Nurs 2017;16:26. 28559745 10.1186/s12912-017-0221-7 PMC5445490

[25] Gillen P , Neill RD, Mallett J et al. Wellbeing and coping of UK nurses, midwives and allied health professionals during COVID-19—a cross-sectional study. PLoS One 2022;17:e0274036. 36129890 10.1371/journal.pone.0274036 PMC9491587

[26] Antonakis J , Bendahan S, Jacquart P, Lalive R. On making causal claims: A review and recommendations. Leadersh Q 2010;21:1086–1120.

[27] Tucker P , Bejerot E, Kecklund G, Aronsson G, Akerstedt T. The impact of work time control on physicians’ sleep and well-being. Appl Ergon 2015;47:109–116. 25479980 10.1016/j.apergo.2014.09.001

[28] Turunen J , Karhula K, Ropponen A et al. The effects of using participatory working time scheduling software on sickness absence: a difference-in-differences study. Int J Nurs Stud 2020;112:103716. 32736788 10.1016/j.ijnurstu.2020.103716

[29] Nijp HH , Beckers DG, Geurts SA, Tucker P, Kompier MA. Systematic review on the association between employee worktime control and work-non-work balance, health and well-being, and job-related outcomes. Scand J Work Environ Health 2012;38:299–313. 22678492 10.5271/sjweh.3307

[30] Dall’Ora C , Ejebu OZ, Griffiths P. Because they’re worth it? A discussion paper on the value of 12-h shifts for hospital nursing. Hum Resour Health 2022;20:36. 35525947 10.1186/s12960-022-00731-2 PMC9077839

